# Surgical outcomes of hepatocellular carcinoma with biliary tumor thrombus: a systematic review

**DOI:** 10.1186/s12876-016-0427-2

**Published:** 2016-01-28

**Authors:** Wenhui Qiao, Feng Yu, Lupeng Wu, Bin Li, Yanming Zhou

**Affiliations:** Department of General Surgery, First Hospital of Lanzhou University, Lanzhou, China; Department of Hepatobiliary Surgery, No.101 Hospital of CPLA, Wuxi, China; Department of Hepato-Biliary-Pancreato-Vascular Surgery, First affiliated Hospital of Xiamen University, Xiamen, China

**Keywords:** Hepatocellular carcinoma, Biliary tumor thrombus, Resection, Prognosis

## Abstract

**Background:**

Hepatocellular carcinoma (HCC) with biliary tumor thrombus (BTT) is rare and its impact on postoperative prognosis remains controversial. The aim of this study was to evaluate the published evidence concerning the outcome of surgical resection of HCC with BTT.

**Methods:**

Eligible studies were identified by searching PubMed and reviewed systematically. Comparisons of the clinicopathologic features and surgical outcomes for HCC patients with or without BTT were analyzed using meta-analytical techniques.

**Results:**

Twenty retrospective studies containing 598 patients that met the selection criteria were included for review. The perioperative mortality was 2.1 % (range, 0–10 %), and the median 5-year overall survival (OS) was 24 % (range, 0–48 %) with a recurrence rate of 63.9 % (range, 42–91 %). Pooled analysis of 13 comparative studies showed that HCC patients with BTT had a higher incidence of vascular invasion (odds ratio [OR]: 4.70, 95 % CI: 2.90–7.60; *P* <0.001), a higher frequency of poor differentiation (OR: 2.07, 95 % CI: 1.23–3.49; *P* = 0.006), and a shorter 5-year OS rate (OR: 0.31, 95 % CI: 0.21–0.64; *P* <0.001) than those without BTT.

**Conclusions:**

Although HCC with BTT has more aggressive biological characteristics and is an indicator of poor prognosis, surgical resection can still provide long-term survival for some patients.

## Background

Hepatocellular carcinoma (HCC) is the fifth most common neoplasm in the world and is the third leading cause of cancer-related death worldwide, with more than 500 000 new cases diagnosed each year [1]. Surgical resection remains the mainstay of curative treatment for this disease. Portal vein thrombus is a frequent event in HCC and has important impact on patient survival after surgical resection [2], while biliary tumor thrombus (BTT) is relatively rare with a reported incidence of 0.53–12.9 % in autopsy and surgical specimens [3–9]. In this regard, few reports are available in the literature addressing the role of surgical resection for this special clinical entity. In addition, the prognostic impact of BTT is controversial [5, 6, 9]. The current study assesses the published literature on surgical resection for HCC with BTT and compares the clinicopathologic features and long-term postoperative outcomes between HCC patients with BTT and those without BTT.

## Methods

### Systematic search strategy

A comprehensive systematic review of all published literature from 1966 to September 2015 was undertaken using PubMed database. The following Medical Subject Headings terms were used: “hepatocellular carcinoma,” “biliary tumor thrombus,” “bile duct tumor thrombus,” and “bile duct thrombus.” Reference lists of all retrieved articles were manually searched to identify further potentially relevant articles. This study was performed according to Preferred Reporting Items for Systematic Reviews and Meta-analyses (PRISMA) guideline guidelines [10].

### Inclusion and exclusion criteria

English language studies with a sample size of at least 10 patients that reported long-term survival data following surgical resection for HCC with BTT were included. Animal studies, letters, reviews, abstracts, editorials, expert opinions, duplicates, studies with fewer than 10 patients, studies involving patients who were treated with nonsurgical management or liver transplantation were excluded.

### Data extraction and quality assessment

Information on study design, first author, country or region, year of publication, study population characteristics, and outcomes of interest were independently extracted by two authors (Yanming Zhou and Feng Yu). Discrepancies between the two reviewers were resolved by discussion and consensus. Study methodology quality was categorized according to the Evidence-based Medicine Levels of Evidence [11].

Macroscopic BTT was defined when it was present in the common hepatic duct or the first to second branches of the bile duct and microscopic BTT was defined when it was present in the third order or more peripheral branches of the bile duct [12].

The primary outcome measures were 1-, 3- and 5-year overall survival (OS) following surgical resection.

### Statistical analysis

Data extracted for BTT group were reported as total and percentage for categorical variables and as median values and range for continuous variables, unless otherwise stated. The results of comparative studies of patients with BTT and without BTT were pooled by the use of Review Manager.software, version 5.1 (The Cochrane Collaboration, Software Update, Oxford). Dichotomous variables were expressed as odds ratio (OR) with a 95 % confidence interval (95 % CI), and continuous variables were expressed as the weighted mean difference (WMD) with a 95 % CI. *χ*^2^ test and I^2^ were used to assess heterogeneity between studies. The random-effect model was used if there was significant heterogeneity (*P* < 0.1); otherwise, the fixed-effect model was used. Publication bias was evaluated via funnel plot. Statistical significance was set at *P* < 0.05.

## Results

### Systematic review

Among 154 potentially relevant papers identified by the initial search, 20 finally met the inclusion criteria in this review and are summarised in Table 1 [3–9, 12–26]. All studies were retrospectively designed, originated from Asia (Japan, *n* = 8 [5, 6, 12, 13, 15, 20, 21, 25]; Mainland China, *n* = 7 [7, 8, 14, 16, 17, 19, 26]; Korea, *n* = 2 [18, 22]; Taiwan, *n* = 1 [9]; India, *n* = 1 [23]; Hong Kong, *n* = 1 [24]) and classified as level-4 evidence. The sample size of each study varied from 13 to 73 patients. Thirteen studies utilised patients without BTT as a control group for comparison [5, 6, 9, 13, 15–17, 19–24].

**Table 1:** Clinical background of included studies

First author (Year)	No.	M/F	Age (years) ^a^	BTT type Ma/Mi	HBsAg n (%)	Anti-HCV n (%)	Cirrhosis n (%)	TS (cm) ^a^	MT n (%)	VI n (%)	PD n (%)	IM n (%)	TCA n (%)
Satoh (2000) [5]	17	15/2	58.2	17/0	5 (29.4)	–	–	–	–	11 (64.8)	5 (29.4)	–	–
Shiomi (2001) [6]	17	15/2	58.8	17/0	7 (46.7)	3 (17.6)	6 (35 %)	6.1	12/16 (75)	13 (76)	0 (0)	5 (29)	–
Peng (2004) [7]	15 ^b^	10/5	49	15/0	13 (86.7)	–	–	5.1	2 (13.3)	5 (33.3)	–	–	–
Qin (2004) [8]	34	28/6	48.5	34/0	34 (100)	–	32 (94.1)	6.4	–	–	–	–	34 (100)
Yeh (2004) [9]	17	14/3	52.3	17/0	–	–	–	3.8	–	12/16 (75)	3/11 (27.2)	5/16 (83.3)	2/11 (18.2)
Esaki (2005) [12]	38	32/6	62	19/19	8 (21.1)	–	10 (26.3)	6.2	19 (50)	27 (71)	11 (28.9)	19 (50)	–
Ikenaga (2009) [13]	15	12/3	66	10/5	4 (27.7)	7 (46.7)	–	5.0	6 (40)	12/14 (85.7)	–	10 (66.7)	2 (13.3)
Luo (2009) [14]	48 ^c^	–	–	48/0	–	–	–	6.2	–	–	–	–	–
Noda (2011) [15]	22	21/1	58	22/0	15 (68.1)	5 (22.7)	6 (27.2)	>5, *n* = 9	20 (90.9)	13 (59)	18 (81.8)	–	–
Shao (2011) [16]	27	24/3	47.1	24/3	26 (96.7)	0 (0)	18 (66.7)	>5, *n* = 10	–	17 (62.9)	26 (96.3)	5 (18.5)	21 (77.7)
Yu (2011) [17]	20^d^	17/3	50.6	20/0	16 (80)	–	14 (70)	4.5	–	12 (60)	13 (65)	4 (20)	15 (75)
Moon (2013) [18]	73	52/21	54.2	73/0	59 (80.8)	2 (2.8)	62 (84.9)	5.8	11 (15)	53 (72.6)	54 (74)	25 (34.2)	–
Meng (2014) [19]	35	24/11	51.3	28/7	26 (74.3)	0 (0)	25 (65.8)	>5, *n* = 24	15 (42.8)	10 (25.8)	10 (25.8)	–	24 (68.6)
Oba (2014) [20]	13	12/1	61	13/0	4 (30.7)	5 (38.5)	3 (23)	4.4	2 (15.4)	12 (92.3)	–	–	–
Kasai (2015) [21]	44	35/9	64	44/0	8 (18.2)	21 (52.5)	14 (31.8)	5.8	16 (36.4)	31 (70.4)	18 (40.9)	–	–
Kim (2015) [22]	31	21/10	53	0/31	26 (83.9)	1 (3.2)	–	4.8	–	28 (90.3)	1 (3.2)	8 (25.8)	7 (22.5)
Rammohan (2015) [23]	39	28/11	52.1	39/0	7 (17.9)	2 (5.1)	Excluded	5.6	–	–	–	–	–
Wong (2015) [24]	37	29/8	57	37/0	30 (81.1)	–	–	6	15 (29.7)	25 (67.6)	11 (29.7)	–	35 (94.6)
Yamamoto (2015) [25]	19	19/0	67	19/0	7 (36.8)	4 (21)	6 (31.5)	4.3	–	–	–	–	–
Zeng (2015) [26]	37	33/4	50	30/7	29 (78.4)	–	33 (89.2)	4.9	9 (24.3)	25 (67.5)	23 (62.2)	6 (16.2)	–

*M* male, *F* female, *Ma* macroscopic, *Mi* microscopic, *HBsAg* hepatitis B surface antigen, *anti-HCV* hepatitis C virus antibody, *ST* solitary tumor, *TS* tumor size, *MT* multiple tumor, *VI* vascular invasion, *PD* poor differentiation, *IM* intrahepatic metastasis, *TCA* tumor capsule absence

^a^ mean or median

^b^ including 1 patients underwent liver transplantation; ^c^ including 5 patients underwent liver transplantation; ^d^ including 2 patients underwent liver transplantation

The included papers described 598 patients who underwent surgical resection for HCC with BTT, including 526 (88 %) patients who had macroscopic BTT and 72 (12 %) patients who had microscopic BTT. Most (80.2 %, 441/550) patients were men. The median or mean age ranged from 47.1 to 60 (median = 54.2) years. Hepatitis B surface antigen (HBsAg) and hepatitis C virus antibody (Anti-HCV) were positive in 60.7 % (324/533) and 14.9 % (50/335) of patients, respectively. Mean or median tumor size ranged from 3.8 to 6.4 (median = 5.4) cm. Cirrhosis, tumor multiplicity, vascular invasion, poor differentiation, intrahepatic metastasis, and tumor capsule absence, occurred in 60.4 % (229/379), 36.8 % (127/345), 67.1 % (306/456), 47.9 % (188/392), 31.6 % (92/291), and 66.7 % (140/210) of patients, respectively.

Table 2 shows the operative intervention and postoperative outcomes. Among 573 patients with available information, hepatectomy with or without tumor thrombectomy was carried out in 423 (73.8 %), followed by hepatectomy combined bile duct resection in 144 (25.1 %), thrombectomy only in 4 (0.7 %), and thrombectomy with hepatic artery ligation and cannulation in 2 (0.35 %). There were 13 perioperative deaths (2.1 %) (range, 0–10 %). The median overall survival was 26.1 months (range, 11.4–47 months). The median 1-, 3- and 5-year OS rates were 72 % (range, 38–93 %), 39 % (range, 11–77 %) and 24 % (range, 0–48 %), respectively. Disease recurrence developed in 64.8 % (310/478) patients.

**Table 2:** Operative intervention and outcomes

References	Hx ± Tb n (%)	Hx + BDR n (%)	Tb only n (%)	Mortality n (%)	Median survival (Months)	Overall survival (%) 1-year 3-years 5-years	Recurrence n (%)
5	12 (70.6)	5 (29.4)	0 (0)	0 (0)	–	58 30 16	10/15 (66.7)
6	12 (70.6)	5 (29.4)	0 (0)	0 (0)	17.6	75 47 28	11 (64.7)
7	7 (50)	4 (28.5)	3 (21.4)	1 (7.1)	14	73 51 21	7 (100)
8	30 (88.2)	1 (2.9)	1 (2.9)	1 (2.9)	–	71 11 –	14/28 (50)
9	–	–	–	1 (5.9)	20.8	60 20 6.7	11/16 (68.7)
12	33 (86.8)	5 (13.2)	0 (0)	0 (0)	31	79 45 33	29 (76.3)
13	12 (80)	3 (20)	0 (0)	0 (0)	11.4	46 23 0	11 (73.3)
14	40 (93)	3 (7)	0 (0)	1 (2.1)	37	93 56 24	10 (21)
15	20 (90.9)	2 (9.1)	0 (0)	0 (0)	–	62 30 30	13/16 (81.2)
16	26 (96.3)	1 (3.7)	0 (0)	1 (3.7)	–	70 26 7.4	25/26 (96.1)
17	12 (66.7)	6 (33.3)	0 (0)	2 (10)	–	73 21 –	7/13 (53.8)
18	42 (57.5)	31 (42.5)	0 (0)	3 (4.1)	–	77 41 32	52/70 (74.3)
19	25 (71.4)	10 (28.6)	0 (0)	0 (0)	19	38 20 11	–
20	7 (53.8)	6 (46.2)	0 (0)	0 (0)	47	92 77 48	6 (46.1)
21	37 (84)	7 (16)	0 (0)	2 (4.5)	23.7	70 38 31	30/41 (73.2)
22	31 (100)	0 (0)	0 (0)	0 (0)	–	90 61 –	20 (64.5)
23	30 (76.9)	9 (23.1)	0 (0)	2 (5.1)	28.6	82 48 10	–
24	9 (24.3)	28 (75.7)	0 (0)	0 (0)	44	69 54 39	23 (62.1)
25	15 (78.9)	4 (21.1)	0 (0)	0 (0)	–	82 39 32	10/15 (66.7)
26	23 (62.2)	14 (37.8)	0 (0)	0 (0)	–	64 24 18	21 (56.8)

*Hx* hepatectomy, *Tb* thrombectomy, *BDR* bile duct resection

### Meta-analysis

A total of 13 comparative studies including 334 patients with BTT and 6361 without BTT were included for analysis [5, 6, 9, 13, 15–17, 19–24]. Table 3 presents a summary of outcomes. Table 3 presents a summary of the outcomes. There was no significant difference in clinicopathological characteristics between the two groups in terms of sex, age, hepatitis viral status (hepatitis B or C virus), presence of cirrhosis, tumor size and multiplicity, tumor capsule absence, and intrahepatic metastasis, while vascular invasion (OR: 4.70, 95 % CI: 2.90–7.60; *P* <0.001) and poor differentiation (OR: 2.07, 95 % CI: 1.23–3.49; *P* = 0.006) were more frequently observed in BTT group than those in non-BTT group.

**Table 3:** Results of meta-analysis comparing hepatocellular carcinoma with or without biliary tumor thrombus

Outcome of interest	No. of studies	No. of patients BTT Non-BTT	OR/WMD	95 % CI	*P* value	HG *P* value
Clinicopathological characteristics						
Male	12	317 5806	1.07	0.97, 1.43	0.67	0.69
Age	9	256 3840	0.01	−1.01, 1.02	0.99	0.01
Hepatitis B surface antigen	11	297 5150	1.33	0.99, 1.79	0.06	0.35
Hepatitis C virus antibody	9	243 4274	0.72	0.37, 1.39	0.33	0.03
Cirrhosis	6	158 3569	0.72	0.51, 1.00	0.05	0.11
Tumor size	7	177 4028	−0.89	−2.63, 0.85	0.32	<0.001
Multiple tumor	6	166 3662	1.85	0.76, 4.52	0.18	<0.001
Tumor capsule absence	7	176 2305	1.26	0.63, 2.51	0.62	0.02
Intrahepatic metastases	5	109 1710	1.47	0.93, 2.33	0.10	0.40
Vscular invasion	12	293 5939	4.70	2.90, 7.60	<0.001	0.001
Poor differentiation	8	227 4003	2.07	1.23, 3.49	0.006	0.05
Long-term outcomes						
1-year overall survival	12	280 4739	0.48	0.31, 0.72	<0.001	0.02
3-year overall survival	12	280 4739	0.45	0.29, 0.68	<0.001	0.007
5-year overall survival	10	231 4021	0.31	0.21, 0.64	<0.001	0.02

*BTT* biliary tumor thrombus, *OR* odds ratio, *WMD* weighted mean difference, *CI* confidence interval, *HG* heterogeneity

**Fig. 1:**
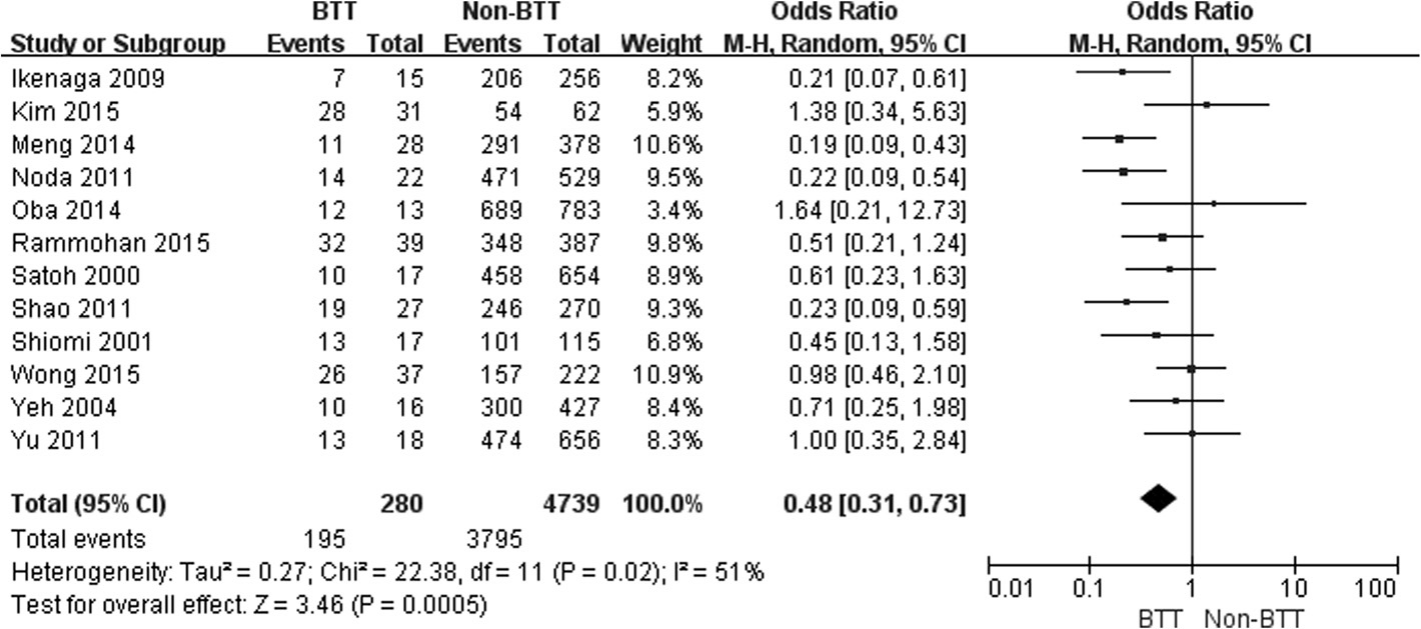
Result of the meta-analysis on 1-year overall survival

**Fig. 2:**
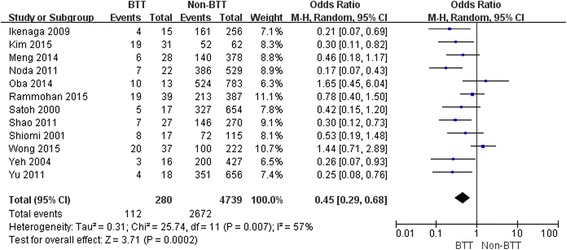
Result of the meta-analysis on 3-year overall survival

**Fig. 3:**
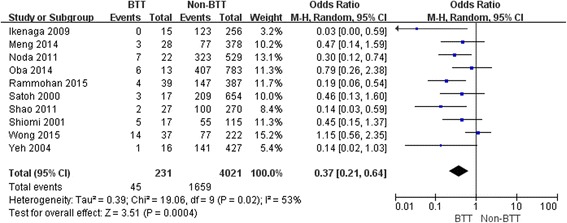
Result of the meta-analysis on 5-year overall survival

The 1-, 3- and 5-year OS rates in patients with BTT were significantly lower than those in patients without BTT (OR: 0.48, 95 % CI: 0.31–0.73; *P* <0.001; OR: 0.45, 95 % CI: 0.29–0.680; *P* <0.001; OR: 0.31, 95 % CI: 0.21 – 0.64; *P* < 0.001, respectively) (Figs. 1, 2 and 3).

**Fig. 4:**
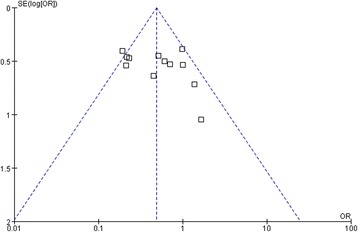
Funnel plot analysis of publication bias. The outcome was the 1-year overall survival

A funnel plot analyzing all studies utilized in the 1-year OS analysis demonstrated asymmetry, suggesting the presence of publication bias (Fig. 4).

## Discussion

HCC patients with BTT usually respond poorly to nonsurgical treatments, such as transcatheter arterial chemoembolization (TACE), percutaneous transhepatic biliary drainage, and radiation. Oba et al. [20] reported that the 1-, 3-, and 5-year OS rate in their 25 patients was 14 %, 5 % and 0 % after nonsurgical treatment, respectively. Luo et al. [14] reported a 5-year OS rate of 0 % for patients treated with TACE (*n* = 27) or biliary decompression (*n* = 40). In this study, patients who received surgical resection had a 5-year OS rate of 24 %, which is far better than the results of the nonsurgical treatments discussed above. So it seems justified to carry out surgical resection for this group of patients.

We found that long-term survival in patients with BTT was significantly shorter than that in patients without BTT. This may be a consequence of the fact that BTT has more aggressive biologically characteristics. As showed in the current study, the incidence of vascular invasion and poor differentiation, two powerful unfavorable prognostic factors, were more frequently observed in BTT group than those in non-BTT group.

The high incidence of postoperative recurrence limits the potential for surgical cure of HCC. Postoperative recurrence is usually classified as early (≤1 year) and late (>1 year) recurrence. Early recurrence was found to be associated with worse prognosis compared with late recurrence.^27^ Ikenaga et al. [13] reported that 53 % of their patients in BTT group developed recurrences in the remnant liver within 3 months after surgery. Qin [8], Noda [15], Shao [16], Zeng [26] and their colleagues reported that more than 50 % of their BTT patients experienced recurrences during the first year after surgery. Shao et al. [16] found that patients with BTT had a higher rate of early recurrence than those without BTT (70.3 % v*s.* 34.8 %; *P* < 0.001). In aggregate, these results suggest that patients with BTT had a higher propensity for early recurrence. It is widely accepted that early recurrence is mainly caused by intrahepatic metastasis from the primary tumor via the venous circulation [27]. As most patients with BTT also had vascular invasion, it is understandable that BTT is likely to recur early after resection. On the other hand, Ikenaga et al. [13] reported two cases of BTT patients without vascular invasion who developed early recurrence in the remnant liver. Similarly, Shao et al. [16] reported nine cases of BTT patients without vascular invasion who experienced early recurrence. These data indicate that dissemination via the bile duct system is another gate of intrahepatic metastasis. In the American Joint Committee on Cancer (AJCC)/International Union Against Cancer (UICC) staging system, information of BTT is not required [28]. In contrast, in the Liver Cancer Study Group of Japan (LCSGJ) staging system, patients with BTT are assigned to the advanced stage and had a less favorable prognosis [29]. Based on the results of the present study, the LCSGJ staging system appears to be more appropriate for HCC lesions than the AJCC/UICC system.

The necessity of bile duct resection for HCC with macroscopic BTT is a subject of debate. Some authors found that BTT rarely invaded the bile duct wall around the hepatic hilus and could be easily removed [5, 6, 15, 25]. Besides, analysis of the OS rate by some studies showed that bile duct resection did not seem to provide any significant benefit [5, 6, 15], and therefore they suggested that such resection should be avoided unless essential for technical purposes. Whereas other authors advocated routine bile duct resection for HCC patients with BTT, knowing that it may minimize recurrence due to better eradication of the microscopic tumor [24, 26]. As all these studies involved the analysis of only a small number of patients, comparisons of the results between the two groups may be of limited value, and therefore future studies with a larger number of patients are required.

There have been relatively few studies in the literature reporting the practice and outcome of liver transplantation for the treatment of HCC with BTT. Peng et al. [7] reported one patient who died of recurrence at 27 months after liver transplantation. Hwang et al. [30] reported a 5-year OS rate of 50 % in a cohort of 14 patients. Although these authors believed that liver transplantation may be a potential treatment option for HCC with BTT, it is prudent to draw any firm conclusion before more results are obtained from further studies with larger sample sizes.

The present study has several limitations. First, all the included studies were performed in the Asia-Pacific region, which may affect the generalizability. In addition, the retrospective nature of eligible studies is vulnerable to introduce potential bias. For example, there were differences in observation periods, disease stages and surgical methods between the institutions. For this reason, significant heterogeneity was tested in the meta-analytic statistical outcomes. Finally, as only 13 of the 20 included studies were eligible for meta-analysis, the impact of BTT on the prognosis may be underestimated.

## Conclusions

HCC with BTT has aggressive biological characteristics and is an indicator of poor prognosis. However, surgical resection can still provide long-term survival for some patients. More effective adjuvant therapies need to be developed to improve the outcome. Adoptive immunotherapy [31], antiviral therapy [32, 33], intrahepatic injection of 131I-lipiodol [34], and sorafenib- or peretinoin-based chemotherapy [35, 36] may provide beneficial effects.
